# Expert Opinions on Postoperative Complications in Breast Cancer Surgery After Neoadjuvant Chemotherapy: A Descriptive Study Through Structured Interviews With Surgeons in Austria

**DOI:** 10.1155/tbj/6589301

**Published:** 2026-05-31

**Authors:** Carmen Leser, Rebecca Eisl, Georg Dorffner, Fiona Kabashi, Daphne Gschwantler-Kaulich, Christine Deutschmann, Sabine Danzinger, Rupert Koller

**Affiliations:** ^1^ Department of Obstetrics and Gynecology, Medical University of Vienna, Vienna, Austria, meduniwien.ac.at; ^2^ Institute for Artificial Intelligence, Medical University of Vienna, Vienna, Austria, meduniwien.ac.at; ^3^ Department of Plastic, Aesthetic and Reconstructive Surgery, Clinic Landstrasse and Clinic Ottakring, Vienna, Austria

**Keywords:** breast cancer surgery, neoadjuvant chemotherapy (NACT), postoperative complications, risk factors, wound healing and bleeding

## Abstract

**Background:**

Neoadjuvant chemotherapy (NACT) is an important component in preparing breast cancer patients for surgery. Its impact on postoperative complications, such as wound infections and bleeding, remains unclear. While most studies show no increase in complication rates, factors such as smoking may elevate risk. Understanding surgeons’ perspectives on bleeding and related influences is therefore essential.

**Methods:**

This study used a questionnaire on bleeding and wound healing. After ethical approval in Vienna and Burgenland, 33 surgeons were recruited. Data were collected between July and December 2022 through interviews or self‐administered questionnaires and analyzed descriptively.

**Results:**

Overall, 63.6% of surgeons reported recognizing NACT‐treated patients intraoperatively. Perceptions of blood loss varied, with some noting no difference and others reporting increased bleeding. The influence of tumor size and smoking was debated, with no clear consensus. Most surgeons did not observe prolonged operative times. Challenges in axillary dissection and sentinel lymph node identification were reported, particularly after NACT.

**Conclusion:**

Surgeons’ views on the impact of NACT in breast surgery vary considerably. These findings highlight the complexity of integrating NACT into surgical practice and the need for further research to improve training, patient counseling, and evidence‐based guidelines.

## 1. Introduction

Breast cancer is a complex disease that often requires a multimodal treatment approach, including neoadjuvant chemotherapy (NACT) followed by surgery. Although NACT is effective in reducing tumor size and facilitating surgical resection, its impact on postoperative complications, particularly wound infection and bleeding, remains a topic of significant interest. Despite major advances in surgery, complications—especially low‐grade ones—are often underreported [1]. Several studies have investigated whether NACT increases postoperative morbidity. Bowen et al. found no significant difference between patients with and without NACT [2], and similar results were reported by Beugels et al. for DIEP flap reconstruction [3] and by Lorentzen et al. [4]. Kumar et al. observed that seroma formation was the most common postoperative complication, followed by superficial and deep surgical site infections [5]. Decker et al. found no general increase but identified risk factors such as smoking, functional dependency, obesity, diabetes, hypertension, and mastectomy [6]. Smoking is also associated with a higher risk of postoperative bleeding. Nordestgaard et al. showed significantly increased transfusion rates among smokers across all surgical specialties [7]. In the reconstructive surgery, the complication rates are higher in implant‐based and autologous procedures [8–11]. Regarding bleeding, Lauk et al. reported negligible incidence after bevacizumab administration when appropriately timed before surgery [12], whereas neoadjuvant immunotherapy in gastric cancer was associated with less intraoperative bleeding and faster recovery [13]. The choice of surgical tools may also influence bleeding; Khaled et al. demonstrated reduced blood loss with ultrasonic shear devices compared to monopolar electrocautery in breast surgery after NACT [14].

These factors underline the importance of understanding the interplay between NACT and surgical outcomes to optimize patient care and refine operative strategies. This study aimed to describe surgeons’ perceptions of bleeding complications during and after breast surgery in relation to NACT, determine whether subjective differences exist depending on prior chemotherapy, and explore additional influences such as smoking.

## 2. Materials and Methods

### 2.1. Study Design

A multiple‐choice questionnaire (Table 1) was used to investigate the effects of NACT on the breast surgery complications.

**TABLE 1:** Demographic and professional characteristics of participants.

Characteristics	*n* (%)
Position	Senior consultant: 6 (18.2%) Junior consultant: 25 (75.8%) Resident: 2 (6.0%)

Specialty	Gynecology: 12 (36.4%) General surgery: 16 (48.4%) Plastic surgery: 5 (15.2%)

Age	< 40 years: 6 (18.2%) 40–59 years: 22 (66.7%) ≥ 60 years: 5 (15.1%)

Total breast surgeries performed	1–100: 6 (18.2%) 101–500: 11 (33.3%) 501–1000: 9 (27.3%) > 1000: 7 (21.2%)

Breast surgeries in last 3 years	1–100: 16 (48.5%) 101–500: 15 (45.5%) > 500: 2 (6.0%)

The questionnaire was tested prior to the main study at several institutions, including the Medical University of Vienna; however, this study represents the first description of the questionnaire in an international journal. It was developed by a senior surgeon at the university and revised twice before the present study.

The main questions concerned bleeding complications during and after surgery. Furthermore, complications such as wound healing, dissection of the axilla, identification of the sentinel lymphatic node, duration of operation, and nicotine use status were addressed. Personal information regarding the participants, such as age, educational level, and experience, was collected to standardize the responses.

### 2.2. Ethics Approval and Consent

This study was approved by the ethics board of the General Hospital of Vienna (1114/2022), and consent was obtained from the region of Burgenland. As ordered by the ethics board, a written informed consent form was established for the use of personal data and signed by all participants.

### 2.3. Participants and Inclusion Criteria

Overall, 33 surgeons from certified breast surgery departments in Vienna and Burgenland participated in this study. The inclusion criteria were as follows: surgeons aged between 27 and 65 years and regularly engaged in a certified breast cancer center.

### 2.4. Recruitment

Recruitment was conducted via email to the heads of departments in different hospitals in the abovementioned regions. Data collection via inventories filled out by surgeons was performed using snowball sampling to identify potential subjects. The heads of the departments were asked to forward an email inviting the relevant surgeons in their respective departments to voluntarily participate in the study. Subsequently, direct contact was established with surgeons who were suitable for participation in the study. A total of 39 surgeons were invited, of whom 33 completed the questionnaire, yielding a response rate of 84.0%.

### 2.5. Data Collection

The interviews were conducted in person, by telephone, or via Zoom (Version 5.0.2; Zoom Video Communications, Inc). However, some participants preferred not to be interviewed and completed the questionnaire by post or e‐mail.

### 2.6. Data Protection and Analysis

All interviews included in the present study were conducted between May 5, 2022, and December 14, 2022. All collected data were handled in accordance with the Austrian national data protection regulations. Data analysis was performed using Microsoft Excel (Version 2311) and IBM SPSS Statistics for Mac OS *X* (Version 22.0; IBM Corp., Armonk, NY, USA). Statistical analyses were conducted using both descriptive and inferential approaches.

Descriptive statistics were used to summarize the participant characteristics and questionnaire responses, providing an overview of the response distributions across items.

In addition, exploratory subgroup analyses were conducted to assess the associations between surgical experience and selected questionnaire responses (e.g., perception of intraoperative blood loss, wound‐healing complications, and difficulty in axillary dissection). Associations between nominal variables were evaluated using cross‐tabulation. Statistical significance was assessed using Pearson’s chi‐square (*χ* ^2^) or Fisher’s exact test, as appropriate, depending on the expected cell counts.

As an additional measure of variability in responses, Shannon entropy (H) was calculated for the selected categorical questionnaire items to quantify the distribution and dispersion of responses. Higher entropy values indicate greater variability in the participants’ answers.

Because each questionnaire item captured a single response per participant (i.e., no multiple raters per case), classical interrater agreement measures such as Cohen’s or Fleiss’ kappa were not applicable. Therefore, Shannon entropy was used to assess response variability for selected binary items (e.g., intraoperative recognition of NACT, perception of wound healing complications, and perceived difficulty of axillary dissection).

## 3. Results

### 3.1. Participant Characteristics

Of the 33 participants, six were senior consultants (18.2%), 25 were junior consultants (75.8%), and two were residents (6.0%). Twelve (36.4%) were gynecologists, 16 (48.4%) were general surgeons, and five (15.2%) of the participants were gynecologists, general surgeons, and plastic surgeons, respectively. Most participants were between 40 and 59 years of age (*n* = 22), while six were younger and five were older. In 2021, 49.6% of Austrian physicians were aged between 35 and 55 years; thus, the participant group can be considered representative of age distribution.

Regarding surgical experience all over, six participants had performed 1–100 breast procedures, 11 had performed 101–500, nine had performed 501–1000, and seven had performed more than 1000. Over the past three years, 16 had performed 1–100 breast procedures, 15 had performed 101–500, and two had performed > 500 procedures.

Table 1 summarizes the demographic and professional characteristics of the participants.

## 4. Recognition of NACT Effects

**FIGURE 1:**
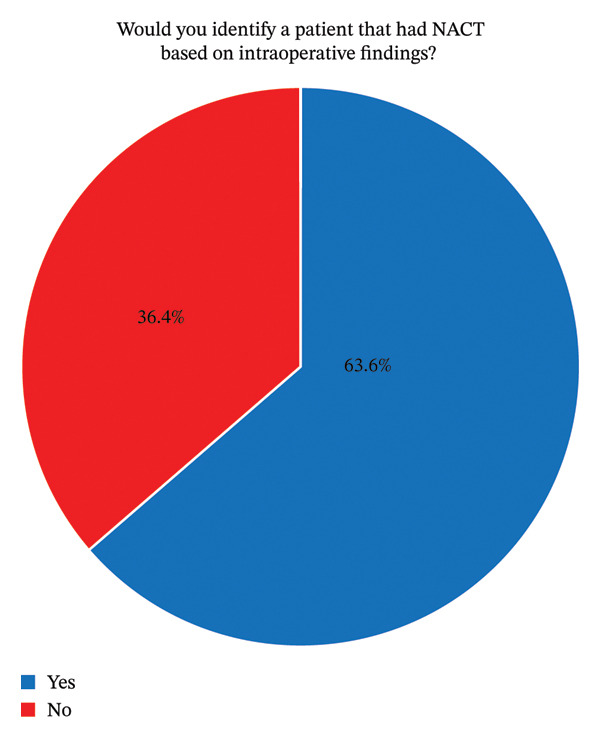
Identification of patients with NACT. This figure illustrates that the majority of the participants would confidently recognize if a patient had undergone NACT based on the intraoperative findings; NACT: neoadjuvant chemotherapy. Identification of patients with prior NACT during surgery. The majority of participants (*n* = 21; 63.6%) reported that they could recognize whether a patient had undergone neoadjuvant chemotherapy (NACT) intraoperatively without reviewing the patient’s records, while 12 participants (36.4%) reported they could not.

When asked whether they could intraoperatively identify whether a patient had undergone NACT without knowing the patient’s history, 21 participants (63.6%) answered “yes” and 12 (36.4%) answered “no” (Figure 1). Among those answering “yes,” nine attributed it to stronger intraoperative bleeding, while others cited changes in perfusion, skin condition, tissue layers, or tissue characteristics. The Shannon entropy for this item was 0.946, indicating a high variability in responses.

**FIGURE 2:**
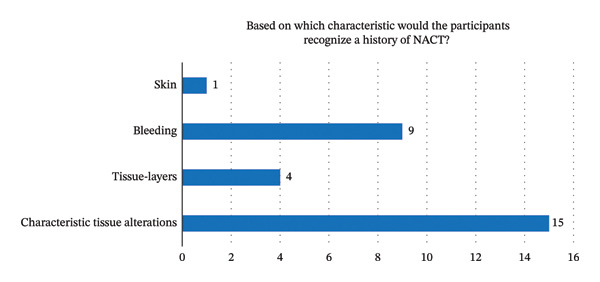
Way of identification of a patient who had undergone NACT. This figure illustrates that a majority of the participants would recognize a history of NACT based on characteristic tissue alterations and higher rate of bleeding; NACT: neoadjuvant chemotherapy. Criteria for intraoperative recognition of prior NACT. Among surgeons who reported the ability to recognize NACT intraoperatively (*n* = 21), the most frequently cited indicators were characteristic tissue alterations, stronger intraoperative bleeding, changes in skin condition, and altered tissue layers. Multiple criteria could be selected.

Most of these surgeons also reported that surgery after chemotherapy was more challenging because of scar tissue formation and anatomical dissection difficulties (Figure 2).

### 4.1. Bleeding Complications

Regarding intraoperative blood loss, 23 participants reported no difference, whereas 10 expected higher bleeding after chemotherapy. There was no statistically significant difference in distribution between surgeons’ experience with bleeding complications (*χ* ^2^ [cF] = 0.01,  *p*  >  0.999\).

**FIGURE 3:**
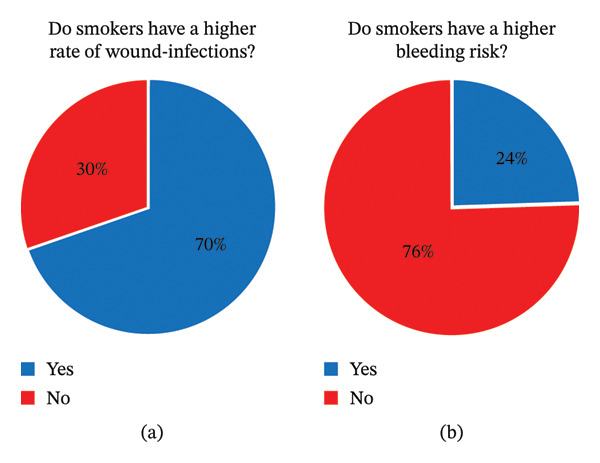
Surgical complications in smokers. The illustrations show that (a) a majority of the participants would expect smokers to have a higher rate of wound infections and (b) 24% would expect smokers to also have a higher risk of blood loss. Perceived influence of NACT and smoking on bleeding and wound healing. Responses indicated variation in perceived intra‐ and postoperative bleeding risk and wound healing complications associated with NACT. In addition, several participants believed smoking increased the risk of these complications, particularly in combination with NACT.

For postoperative bleeding, 25 reported no difference, seven anticipated higher bleeding, and one reported less bleeding after NACT. Twenty‐two ruled out influence of tumor size on bleeding risk, whereas 11 believed that larger tumors were associated with greater bleeding. There was no statistically significant difference in distribution between surgeons’ experience with tumor size bleeding risk (*χ* ^2^ [cF] = 0.06, *p* > 0.999) (Figure 3).

#### 4.1.1. Wound Healing Complications

When asked about postoperative wound complications, 22 participants perceived no difference between patients with or without chemotherapy, while 11 reported more complications after NACT. The Shannon entropy for this item was 0.918, indicating high variability in responses, and no statistical significance was observed (*χ* ^2^ [df = 1] = 0.24, *p* = 0.622). For perceived differences in postoperative wound‐healing complications associated with NACT (11 “yes” vs. 22 “no”), as shown in Table 2.

**TABLE 2:** Frequencies and proportions (row percentages) of reported NACT‐related complications considering surgery experience.

Experience group	No (no difference)	Yes (higher risk)	Row total
≤ 500 surgeries	12 (70.6%)	5 (29.4%)	17
> 500 surgeries	10 (62.5%)	6 (37.5%)	16
Column totals	22 (66.7%)	11 (33.3%)	33

Twenty‐three participants (69.7%) stated that smokers who received NACT had a higher risk of wound‐related complications**.** No statistically significant difference between less experienced and more experienced surgeons was observed (*χ* ^2^ [c.F., corrected by Fisher’s exact test] = 0.41, *p* = 0.708)**.** Less experienced surgeons (≤ 500 lifetime breast surgeries) more often reported increased wound healing complications (47.1%) than more experienced surgeons (> 500 surgeries, 18.8%); however, this difference did not reach statistical significance (*χ* ^2^[df = 1] = 2.97, *p* = 0.085), indicating a comparable distribution difference of reported wound healing complications considering the two experience levels, as shown in Table 3.

**TABLE 3:** Frequencies and proportions (row percentages) of reported wound healing complications considering surgery experience.

Experience group	No	Yes	Row total
≤ 500 surgeries	9 (52.9%)	8 (47.1%)	17
> 500 surgeries	13 (81.3%)	3 (18.8%)	16
Column totals	22 (66.7%)	11 (33.3%)	33

## 5. Operation Time

Regarding the duration of surgery, 10 surgeons felt that NACT prolonged the procedure, while 23 (69,7%) noted no difference. A statistically nonsignificant distribution difference between less experienced surgeons and more experienced surgeons was observed (*χ* ^2^ [c.F.] = 1.96, *p* = 0.259).

### 5.1. Axillary Dissection and Sentinel Lymph Node Identification

Axillary dissection was considered more difficult after NACT by 24 participants, of whom 14 had performed ≤ 500 breast procedures and 10 had performed > 500. Less experienced surgeons were therefore more likely to perceive greater difficulty (82.4% vs. 62.5%); however, this difference was not statistically significant (*χ* ^2^ = 0.79, *p* = 0.374).

Chi‐square testing revealed a nonstatistically significant association between lower and higher experience perceived difficulty in lymph node identification (*χ* ^2^ [df = 1] = 1.73, *p* = 0.188). The findings provide no evidence that experience is a significant determinant of perception.

**FIGURE 4:**
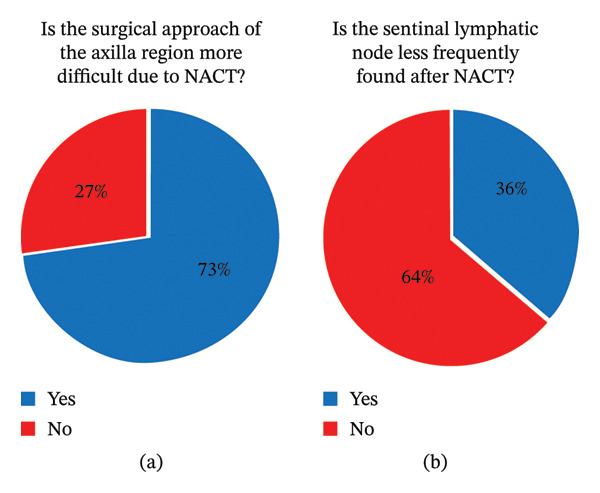
Identification of Sentinel Lymphatic Node. The figure illustrates that (a) most participants judged that surgery of the axilla was more difficult after prior NACT; however, (b) the majority of the participants were still confident to identify the sentinel lymphatic node; NACT: neoadjuvant chemotherapy. Sentinel lymph node identification after NACT. Methods used for sentinel lymph node detection included radioactive markers alone, a combination of radioactive markers and blue dye, or blue dye alone. A total of 12 participants (36.4%) reported that sentinel lymph nodes were less frequently identified in patients who had undergone NACT.

Sentinel lymph node detection was performed using radioactive markers alone (*n* = 18), a combination of markers and blue dye (*n* = 9), or blue dye alone (*n* = 6). Patent blue and methylene blue were used as dyes. Twelve participants reported lower sentinel lymph node detection rates after NACT (Figure 4).

Table 4 provides a detailed summary of the main descriptive results of the questionnaire.

**TABLE 4:** Summary of main questionnaire responses regarding NACT‐related surgical outcomes.

Question	Response
Can identify NACT intraoperatively	Yes: 21 (63.6%) No: 12 (36.4%)

Basis for recognition (multiple answers possible)	Stronger bleeding: 9 tissue alterations: multiple skin changes: multiple

Intraoperative bleeding after NACT	More: 10 (30.3%) no difference: 23 (69.7%) less: 0

Postoperative bleeding after NACT	More: 7 (21.2%) no difference: 25 (75.8%) less: 1 (3.0%)

Tumor size influence on bleeding	Yes: 11 (33.3%) No: 22 (66.7%)

Higher bleeding risk in smokers	Yes: 8 (24.2%) No: 25 (75.8%)

More wound complications after NACT	Yes: 11 (33.3%) No: 22 (66.7%)

Higher wound complication risk in smokers with NACT	Yes: 23 (69.7%) No: 10 (30.3%)

Operation time after NACT	Longer: 10 (30.3%) No difference: 23 (69.7%)

Axillary dissection more difficult after NACT	Yes: 24 (72.7%) No: 9 (27.3%)

Sentinel node less frequently identified after NACT	Yess: 12 (36.4%) No: 21 (63.6%)

Variability analysis showed an entropy value of 0.946 for intraoperative recognition of NACT (21 “yes” vs. 12 “no”), indicating a higher degree (63.6%) of disagreement among the participants (Table 5).

**TABLE 5:** Questionnaire used in the present study with questions related to (A) the participants and (B) surgical outcomes.

Category	Question	Responses
A1	Age?	() < 40
		() 40–49
		() 50–59
		() > 60

A2.1	Level of expertise?	() Trainee
		() Consultant
		() Senior consultant

A2.2	Field of expertise?	() Gynecology
		() General surgery
		() Plastic surgery
		() Others ……………………

A2.3	Position in hospital?	() Surgical Resident
		() Consultant
		() Senior consultant
		() Chief‐surgeon
		() Others ……………………

A3.1	Which hospital?	……………………

A4	How many breast‐surgeries overall?	() 1–100
		() 101–500
		() 501–1000
		() > 1000

A5	How many breast‐surgeries in last 3 years?	() 1–100
		() 101–500
		() > 500

A6	Which kind of procedures performed?	[] Breast‐conserving surgery
		[] Ablation
		[] Oncoplastic surgery
		[] Others

B1	Would you notice if your patient had had a NACT	() Yes
		() No

B1.1	If yes, due to which characteristic?	[] Characteristic tissue alterations
		[] Tissue‐layers
		[] Bleeding
		[] Characteristics of the skin

B2	Intra‐ and postoperative blood‐loss after NACT is	() More
		() Unchanged
		() Less

B3	Does the tumor‐size have any impact on the blood‐loss?	() Yes
		() No

B4	Postoperative problems with wound‐healing after NACT are	() More
		() Unchanged
		() Less

B5	Do smokers have a higher risk of blood‐loss after NACT?	() Yes
		() No

B6	Do smokers have a higher risk of postoperative wound‐healing problems after NACT?	() Yes
		() No

B7	Is the preparation of the axilla more difficult after NACT?	() Yes
		() No

B8	How often is the sentinel‐lymphatic node identified?	%

B8.1	How is the sentinel‐lymphatic node identified?	[] Blue‐dye
		[] Radioactive marker

B8.2	Is the sentinel‐lymphatic node less frequently found after NACT?	() Yes
		() No

B9	The time of operation after NACT is	() Longer
		() Unchanged
		() Shorter

B10	Other remarks	

*Note:* NACT: neoadjuvant chemotherapy.

For the perceived difficulty of axillary dissection after NACT (24 “yes” vs. 9 “no”), the entropy was 0.845, indicating lower variability than the other two parameters. These results suggest that surgeons’ perceptions varied widely on some topics (e.g., recognition of NACT) but were relatively consistent on others (e.g., axillary dissection difficulty).

## 6. Discussion

In this exploratory study, we investigated surgeons’ perspectives on the influence of NACT on intra‐ and postoperative complications in breast surgery, utilizing a standardized multiple‐choice questionnaire [15–17]. Such questionnaires provide a structured and reproducible means of gathering information across respondents and have been widely used to assess surgical knowledge, technical experience, and research competence. However, this method is inherently subjective, as it relies on surgeons’ interpretations of the questions and their prior clinical experience. Our findings revealed considerable diversity in the surgeons’ ability to recognize the intraoperative effects of NACT. Most (63.6%) respondents reported being able to identify whether a patient had undergone NACT without prior information primarily due to stronger intraoperative bleeding or changes in tissue characteristics, skin quality, or perfusion. This supports previous studies suggesting that NACT can produce noticeable intraoperative changes, such as altered tissue planes or increased fibrosis, which may influence surgical technique and preparation [18]. A key observation was the perception that surgery after NACT is more technically demanding, particularly because of scar tissue formation and challenging anatomical dissections. While most participants reported no difference in overall operation time, those perceiving increased difficulty were more often less‐experienced surgeons (≤ 500 lifetime breast surgeries). Although this difference did not reach statistical significance (*χ* ^2^ = 0.79, *p* = 0.37), it suggests that surgical experience may mitigate perceived challenges. Similar findings have been described in the literature, where NACT was not associated with a higher overall rate of postoperative complications in immediate breast reconstruction (IBR) but was linked to increased implant or expander loss [19], and no significant increase in surgical site infections was reported [20]. Perceptions of intraoperative and postoperative bleeding varied. Approximately, one‐third of the participants anticipated greater blood loss after NACT, whereas the majority did not expect a difference. Opinions also diverged regarding the influence of tumor size and smoking status. Certain chemotherapeutic agents, particularly platinum‐based regimens used in triple‐negative breast cancer, are known to cause thrombocytopenia, which may increase the risk of bleeding during and after surgery [19]. Furthermore, surgical complexity and the extent of resection may contribute to variability in bleeding risk [21]. Postoperative wound healing complications were another area of heterogeneous responses. While two‐thirds of the respondents reported no difference between NACT and non‐NACT patients, one‐third perceived more wound complications after NACT. Notably, less experienced surgeons more often reported such complications (47.1% vs. 18.8%), although this was not statistically significant (*χ* ^2^ = 1.84, *p* = 0.18). This perception aligns with the reports of delayed healing and increased wound infections after NACT [22], whereas other studies have reported low complication rates [5, 6]. Axillary dissection was reported as more difficult after NACT by 72.7% of participants, with this perception significantly associated with lower experience levels (*χ* ^2^ = 5.23, *p* = 0.022). This is consistent with the findings that chemotherapy‐induced tissue changes, such as fibrosis, can obscure anatomical landmarks and increase technical difficulty. Despite this, sentinel lymph node detection was generally maintained, although 36.0% of participants reported a reduced detection rate after NACT, in line with the literature suggesting that chemotherapy can alter lymphatic mapping and tracer distribution [21]. Taken together, these results indicate that while objective complication rates after NACT may not be substantially higher according to the broader literature [18–20], subjective surgical experience—particularly in relation to technical difficulty, tissue handling, and wound healing—varies considerably among surgeons. These differences appear to be influenced by surgeon experience and underscore the importance of tailored surgical strategies, multidisciplinary coordination, and integration of NACT‐specific considerations into surgical training.

The limitations of the study conclude the small sample size and the use of a questionnaire, which is reported for the first time in an international journal. Another limitation is the restricted generalizability, as the surgeons surveyed from the three Viennese clinics do not represent the whole of Austria. Accordingly, a selection bias may be assumed. This study is subject to the inherent limitations of self‐reported data, including potential recall bias. As no objective cross‐validation with actual patient outcomes was performed, future studies integrating clinical outcome data may help further validate surgeons’ perceptions.

In conclusion, our study provides new insights into how surgeons perceive and adapt to the challenges of breast surgery after NACT. While objective complication rates appear to remain relatively stable in the literature, subjective factors such as technical complexity, tissue characteristics, and wound healing continue to influence surgical decision‐making. Future research should combine subjective assessments with prospective clinical data to determine how NACT influences surgical outcomes and inform evidence‐based guidelines that reflect both clinical and experiential perspectives [23–25].

## Author Contributions

All authors meet the ICMJE criteria. Some authors participated directly in this study. This dual involvement has the potential to introduce bias in several aspects of the research process, including the study design, data collection, data analysis, and interpretation of results.

Carmen Leser: writing original draft.

Rebecca Eisl: data collection.

Georg Dorffner: formal analysis.

Fiona Kabashi: revised draft.

Daphne Gschwantler‐Kaulich: proof reading and methodology.

Christine Deutschmann: data collection.

Sabine Danzinger: proof reading and revised draft.

Rupert Koller: methodology.

## Funding

No funding was received for this manuscript.

## Disclosure

The abstract of this study was presented at the Austrian congress of senology 2024.

## Ethics Statement

All procedures involving human participants performed in this study were in accordance with the ethical standards of the Institutional Research Committee and with the 1964 Declaration of Helsinki and its later amendments. Informed consent was obtained from all the participants.

This declaration aims to assure the research community and public of their commitment to the ethical conduct and integrity of our research.

The authors disclose this potential conflict of interest to ensure transparency and to uphold the ethical integrity of the study.

## Conflicts of Interest

The authors declare no conflicts of interest.

## Data Availability

The data that support the findings of this study are available from the corresponding author upon reasonable request.
